# Development of a cDNA microarray for the measurement of gene expression in the sheep scab mite *Psoroptes ovis*

**DOI:** 10.1186/1756-3305-5-30

**Published:** 2012-02-08

**Authors:** Stewart TG Burgess, Alison Downing, Craig A Watkins, Edward J Marr, Alasdair J Nisbet, Fiona Kenyon, Carol McNair, John F Huntley

**Affiliations:** 1 Moredun Research Institute, Pentlands Science Park, Bush Loan, Edinburgh. Midlothian. EH26 0PZ. UK; 2 ARK-Genomics, The Roslin Institute, University of Edinburgh, Roslin. Midlothian. EH25 9PS, UK; 3 Strathclyde Institute of Pharmacy and Biomedical Sciences, University of Strathclyde, 161 Cathedral Street, Glasgow. G4 0RE. UK

**Keywords:** *Psoroptes *, sheep, microarray, gene, expression

## Abstract

**Background:**

Sheep scab is caused by the ectoparasitic mite *Psoroptes ovis *which initiates a profound cutaneous inflammatory response, leading to the development of the skin lesions which are characteristic of the disease. Existing control strategies rely upon injectable endectocides and acaricidal dips but concerns over residues, eco-toxicity and the development of acaricide resistance limit the sustainability of this approach. In order to identify alternative means of disease control, a deeper understanding of both the parasite and its interaction with the host are required.

**Methods:**

Herein we describe the development and utilisation of an annotated *P. ovis *cDNA microarray containing 3,456 elements for the measurement of gene expression in this economically important ectoparasite. The array consists of 981 *P. ovis *EST sequences printed in triplicate along with 513 control elements. Array performance was validated through the analysis of gene expression differences between fed and starved *P. ovis *mites.

**Results:**

Sequences represented on the array include homologues of major house dust mite allergens and tick salivary proteins, along with factors potentially involved in mite reproduction and xenobiotic metabolism. In order to validate the performance of this unique resource under biological conditions we used the array to analyse gene expression differences between fed and starved *P. ovis *mites. These analyses identified a number of house dust mite allergen homologues up-regulated in fed mites and *P. ovis *transcripts involved in stress responses, autophagy and chemosensory perception up-regulated in starved mites.

**Conclusion:**

The *P. ovis *cDNA microarray described here has been shown to be both robust and reproducible and will enable future studies to analyse gene expression in this important ectoparasite.

## Background

Sheep scab, a highly contagious disease caused by the mite *Psoroptes ovis*, is characterised by pruritis and irritation of host skin and is therefore a major welfare concern in addition to the substantial costs associated with lost performance, preventative measures, and treatment [1,2]. Current disease control strategies are heavily reliant upon injectable endectocides and acaricidal dips but concerns over residues, environmental contamination and the development of acaricide resistance limit the sustainability of this approach and have resulted in growing interest in the development of alternative control methods [3]. The development of such methods requires a more detailed understanding of both the parasite and its interaction with the host.

The basic biology of the mite is well understood: The entire life cycle of *P. ovis *is carried out on the ovine host and takes from 11-19 days from egg hatch to egg production by the adult [4]. Mites can survive off-host, enabling their transfer from animal to animal; however, they only remain infective for 15-16 days once removed from the skin [5]. *P. ovis *is a non-burrowing, surface exudate feeder capable of consuming serous fluids, lymph and red blood cells [6]. Mites survive on the surface of the skin and their mouthparts, which are thought to abrade rather than pierce the skin, do not penetrate beyond the stratum corneum, the outermost layer of the skin [7]. The available evidence suggests that mites abrade the stratum corneum and deposit allergens as they progress. The combination of mechanical skin abrasion, mite allergen deposition and grooming behaviour by the host in response to the pruritis caused by the mites triggers the subsequent activation of a cutaneous inflammatory response [8,9] including an exudate which has been proposed to supply the mite with a food source [10]. Establishment of a *P. ovis *infestation is therefore the result of a complex interaction between the host and the mite, during which the mite appears to initiate reactions conducive to its own establishment and maintenance [11]. The skin lesions are induced by mite-derived pro-inflammatory factors, a likely source of which are mite excretory/secretory products, including potent enzymes and allergens (reviewed in [10]). While several mite products have been identified, including enzymes and homologues of allergens of the house dust mite (HDM), *Dermatophagoides pteronyssinus *and the scabies mite *Sarcoptes scabiei*, their functions in disease progression remain largely unknown [12].

In the host, a major histological feature of sheep scab is the rapid (within 24 hours) epidermal influx of eosinophils and neutrophils, followed by blister formation and a pronounced serous fluid exudate resulting in dermal oedema [13]. Prior infestation with sheep scab mites alters the progression of subsequent infestations and reductions in lesion size have been observed in secondary infestations in sheep [14]. This evidence points to the development of a protective immune response to *P. ovis *in sheep and offers encouragement for control by vaccination [3]. Vaccination with fractionated *P. ovis *soluble extracts has resulted in a 15-fold reduction in mite numbers and a 4-fold reduction in lesion size [15,16]. However, identification of the individual proteins involved in the development of this partially protective immunity has not yet been achieved. To further understand the mite:host interaction and identify suitable avenues for the discovery of novel interventions we have previously generated a *P. ovis *cDNA library and undertaken an expressed sequence tag (EST) approach [17]. This paper describes the use of these ESTs in the construction of a *P. ovis *cDNA microarray and the utilisation of this array, the first resource of its type for this economically important ectoparasite, under biological conditions to analyse mite gene expression during exposure to, and removal from, the host. This approach was used to analyse which mite genes were expressed at enhanced levels during critical events in the host parasite relationship, for example mite feeding and digestion - processes which are vital to parasite survival.

## Methods

### *Psoroptes ovis *mite collection

Ethical approval for this study was obtained from the Moredun Research Institute Experiments Committee. *P. ovis *mites (a mixed population consisting of adults, nymphs and larvae) were harvested from infested donor animals maintained at the Moredun Research Institute as previously described [8]. For the construction of the *P. ovis *cDNA library ~800 mg of live mites were snap frozen in liquid nitrogen and stored at -80°C for subsequent RNA extraction. This stock of *P. ovis *mite RNA was used in the construction of the mite cDNA library and also represented the "fed" mite sample. "Starved" mites were obtained by following the same procedure as above, except that, following harvesting, mites (~100 mg) were placed into a 75 cm^2 ^vented cap cell culture flask (Corning, UK) and incubated for 4 days at 25°C with 80-90% relative humidity.

### Extraction of total RNA from *P. ovis*

Total RNA was isolated from mites as described previously [18]. RNA samples were further purified using a Qiagen RNeasy kit, following the manufacturer's RNA cleanup protocol and on-column DNase I digestion for 15 minutes at room temperature, before elution into RNase free dH_2_O. Total RNA yield was assessed using a Nanodrop spectrophotometer and RNA quality was assessed using an Agilent Bioanalyser (Agilent, UK).

### Construction and sequencing of a *P. ovis *mite cDNA library

A normalised *P. ovis *cDNA library was constructed by Eurofins MWG, Germany from total RNA extracted as above. The cloning vector used was pBluescript II sk+ and the primary library titre was estimated to be 1,150 cfu/μl; the total number of clones was estimated to be 2.3 million and the average insert size was ~0.9 kb. Normalisation was carried out by denaturation and subsequent reassociation of the cDNA strands. Nucleotide sequence was obtained from 1574 randomly selected clones (Eurofins MWG, Germany). ESTs with an overlap of > 50 bp were assembled into 165 contiguous sequences (contigs) while the remaining 1,091 ESTs remained as singletons. ESTs were filtered to remove poor quality sequences (EST length < 100 bp), to remove vector and adaptor sequence and to mask polyA tails. In order to identify homologues of the assembled *P. ovis *EST sequences, all singletons and contigs were BLAST searched against the Genbank non-redundant nucleotide and amino acid databases using BLASTn and BLASTx, respectively [19]. Sequences with homologue descriptions containing the keywords ribosomal, tRNA, mitochondrial and *Escherichia coli *were removed, leaving 1,033 ESTs for subsequent polymerase chain reaction (PCR) amplification.

### Amplification of cloned *P. ovis *cDNAs

*P. ovis *amplicons, representing the EST sequences (n = 1,033) described above were amplified by PCR using vector-specific oligonucleotide primers in the following reaction for each 384 well plate (3 plates in total used): 168 μl FastStart Taq polymerase (Roche, UK); 15,378 μl dH_2_O; 2,100 μl 10× Buffer (Roche, UK); 2,100 μl 2 mM dNTPs (Roche, UK); 1,260 μl 25 mM MgCl (Roche, UK); 42 μl BSKS F primer (100 pmol/μl) and 42 μl BSKS R primer (100 pmol/μl) (ARK Genomics, UK) for a total PCR reaction volume of 50 μl per well (reactions carried out in 96-well plates). PCR amplification was performed in a Dyad PCR machine (MJ Research, UK) and started with incubation at 95°C for 6 mins followed by 15 cycles of 95°C for 30 secs; 58°C for 30 secs and 72°C for 1 min. Amplification then progressed for a further 19 cycles with the same conditions except that the 72°C product extension phase was extended by 5 seconds after each cycle. PCR amplification was concluded with 7 min incubation at 72°C. PCR product cleanup was performed using a Sciclone Liquid Handling System (Caliper, UK). Each 50 μl PCR reaction was filtered through a 384-well PCR filter plate (Millipore, UK) using positive head pressure for 10 mins, washed with 100 μl of MilliQ water and positive pressure applied for a further 10 minutes. Forty microlitres of dH_2_O was added to each well and mixed with shaking for 10 mins, samples were then aspirated with mixing and transferred to a 384-well cleanup plate (Genetix, UK). Successful PCR product amplification was assessed by the presence of a single product band through gel electrophoresis; 2 μl PCR product was mixed with 7 μl TE/glycerol (2:1) mix and 5 μl loaded onto a 1% agarose gel. PCR amplification yield was assessed using a Fluoroskan Ascent microplate fluorometer (Thermo Fisher, UK). Briefly, PCR product concentration was assessed by adding 20 μl of a 1:200 dilution in TE of each cleaned PCR product into a 384-well Fluorotrac plate (Greiner, UK) along with 20 μl Picogreen solution (Thermo Fisher, UK) and DNA concentration determined by comparison to a standard curve. Fifty two of the 1,033 EST sequences failed to sufficiently amplify either a single PCR product or a sufficient yield of product for printing and were therefore excluded from the microarray generation, leaving 981 ESTs represented on the *P. ovis *cDNA microarray.

### EST annotation

The 981 *P. ovis *ESTs used for printing the cDNA microarray were annotated by homology comparison employing the NCBI non-redundant (nr) database using the NCBI BLASTn function within the blast2go software package [20-22]. Where available, Gene Ontology (GO) annotation was also associated with each probe from within the blast2go package. This process and the annotated dataset has been published previously [17] and annotation of the *P. ovis *cDNA microarray is available in Additional File 1.

### Construction of the *P. ovis *cDNA microarray

In order to obtain representative data for the relative expression of each probe and reduce overall variation, all EST probes were printed onto the arrays in triplicate. To aid data analysis and interpretation, a number of standard control probes were also printed, including Landing Lights for array alignment, salmon sperm DNA, Cot-1 DNA, *P. ovis *and murine genomic DNA probes and a range of positive and negative spike-in control probes (Alien Spot Report, Stratagene, UK). Both positive and negative control probes were printed onto the array in a minimum of triplicate. Array printing was performed on a Super Marathon microarray Inkjet printer (Arrayjet, UK) using a 32 JetSpyder to pick up DNA samples from the plate and transfer to the inkjet print head. Slides used were GAPSII (Corning, UK). All test probes were printed at 115 ng/μl using a print volume of 14 μl. Alien Spot Report probes were printed at either 100 ng/μl or in a dilution series of 8 spots (100 ng/μl - 10 fg/μl). Human Cot-1 DNA and Salmon Sperm DNA controls were printed at 50 ng/μl, polyA control probe was printed at 5 ng/μl whilst *P. ovis *and murine genomic DNA were printed at 150 ng/μl. After printing, slides were baked at 80°C for 4 hours to allow covalent bond formation, fixing the cDNA to the slide. Slides were then blocked with bovine serum albumin (BSA, 0.1 mg/ml in dH_2_O) at 42°C for 1 hour to prevent non-specific binding.

Arrays were printed, four on each slide, to fit within the confines of the MAUI (A4 mixer) 4× hybridisation cassette (BioMicro Systems, USA) allowing 4 arrays to be run on a single slide. Each array consisted of 3 replicate blocks of 36 × 32, arranged 12 blocks per slide for the 4 arrays. Each slide contained a total of 13,824 probes, with each of the 4 arrays consisting of 3,456 probes. This was broken down into 2,943 *P. ovis *probes (981 ESTs printed in triplicate) and 513 control elements which included 15 positional controls with 5 probes per block [Landing Lights, Alien Spot Report (Stratagene, UK) PCR product 1], 9 *P. ovis *genomic DNA (gDNA) probes, 9 mouse gDNA probes, 3 polyA control probes, 3 Salmon Sperm DNA control probes, 3 cot-1 DNA probes, 330 spotting buffer probes, 96 positive spike-in controls printed as 4 sets of 8 probes in a dilution series in triplicate (Alien Spot Report PCR products 7-10) and 45 negative spike-in controls printed as 5 sets of 9 probes (Alien Spot Report PCR products 2-6). The *P. ovis *cDNA microarray and the associated GAL file have been submitted to the ArrayExpress database and are accessible under the following accession number: A-MEXP-2063, with the title: *Psoroptes ovis *cDNA microarray.

### Microarray quality control

To limit the degree of slide to slide variation for array analysis, the *P. ovis *cDNA arrays were printed in a single print run. Array print quality was checked by randomly selecting 4 microarray slides from across the print run and hybridising with Panomer-9 oligonucleotides to visualise both the shape and size of all printed spots, ensuring uniformity in the amount of DNA spotted. This analysis confirmed that the microarray printing was of a consistent and acceptable quality (Data not shown).

### RNA preparation, labelling and amplification

Linearly amplified RNA (aRNA) was generated from 100 ng total RNA of each sample for analysis using the MessageAmp aRNA kit (Ambion, UK) according to the manufacturer's instructions. The aRNA was indirectly coupled with a fluorescent Cy dye, either Cy3 or Cy5 (GE Bioscience, UK) by the incorporation of a 5-(3-aminoallyl)-UTP in a cDNA synthesis reaction, followed by dye binding and purification following the protocol available on the ARK Genomics website (http://www.ark-genomics.org/protocols/DyeCouplingAaRNA.php).

### Microarray hybridisation and washing

Hybridisations were performed on a MAUI microarray hybridisation system (BioMicro Systems, USA) using the MAUI Mixer A4 hybridisation cassette with a 16 μl fill volume per chamber (BioMicro Systems, USA) which allowed the four microarrays on a single slide to be treated independently. The hybridisation mixture consisted of the following: 13.6 μl of a 1:1 mixture of UltraHyb (Ambion, UK) and 2 × SSC (Sigma, UK); 1.6 μl PolyA (10 mg/ml, Sigma, UK); 1.6 μl of 25 pmol Cy3 labelled sample; 1.6 μl of 25 pmol Cy5 labelled samples; 0.8 μl Salmon Sperm DNA (10 mg/ml, Invitrogen, UK) and 0.8 μl BSA (50 mg/ml, Ambion, UK) to a final volume of 20 μl. Hybridisation was performed at 42°C for 16 hours in a microarray hybridisation oven (Agilent Technologies, UK). Slides were washed by dipping up and down for 1 min in Buffer 1 (0.2× SSC + 0.5% SDS) and then for one min in Buffer 2 (0.2× SSC). Slides were then placed into slide racks with lint free tissue at the bottom, and centrifuged at 1,200 rpm for 6 mins to dry.

### Microarray scanning, data extraction and data analysis

Slides were scanned on an Axon 4200AL microarray scanner (Molecular Devices, UK) with laser power set at 80%. The optimum fluorescent signal with limited saturation was selected using the Auto PMT setting. Data extraction was performed within GenePix Pro (Version 7, Molecular Devices) for each individual array using the GenePix Array List (GAL) file. Data analysis was performed using the Partek Genomics Suite (Partek Inc, USA). Dye swaps were performed for all of the array comparisons and raw microarray data for each array were normalised using the default RMA function within the software and differential expression of transcripts represented by the probes determined using the one way analysis of variance (ANOVA) function. The batch effect ANOVA function of Partek was run to remove the effect of dye from the results. Multiple test correction was performed using the Benjamini & Hochberg False Discovery Rate (FDR) procedure with an FDR corrected p-value cut-off of ≤0.05 [23].

### Scatter plot analysis of microarray performance

In order to determine the ability of the array to detect differences in gene expression under experimental conditions a range of hybridisations were performed comparing control (fed *P. ovis *RNA) and biological (starved and fed *P*. ovis RNA) samples. Scatter plots were prepared using the raw non-normalised, transformed (natural log) array data, averaged for each comparison from the four hybridisations (2 dye-swapped (Cy3/Cy5) replicates each) and linear regression analysis performed to determine the degree of variance between the individual comparisons using GraphPad Prism (GraphPad Software Inc, Version 5.04).

### Quantitative real-time PCR (qPCR) analysis of selected microarray probes

qPCR analysis was performed on the original RNA samples used to generate the aRNA employed in the microarray studies, i.e. control (fed) *P. ovis *and starved *P. ovis *mite RNA. Two differentially expressed candidate genes from the fed vs starved *P. ovis *microarray comparison were selected for further analysis by qPCR, namely Pso o 1 and Pso o 2. Gene specific primers were designed based on the *P. ovis *sequence data for Pso o 1 and Pso o 2 using the Primer3 program [24]. Primer sequences were as follows: Pso o 1-Forward (5'TCAAGCTTGCCAAATCGGCGC'3), Pso o 1-Reverse (5'CACCACCGCAACCGTGTTGTG'3), Pso o 2-Forward (5'AGGCTGTTCAGGTGATTACTGCGT'3), Pso o 2-Reverse (5'TGGCAACCATCATGATCACGCCA'3). Relative quantification of gene expression was performed using the standard curve method and data was normalised to the level of a housekeeping gene [*P. ovis *beta-actin, primer sequences as follows: beta-actin-Forward (5'TGAATTGCCTGATGGTCAAG'3), beta-actin-Reverse (5'TGGCGAACAAGTCTTTACGG'3)]. First strand cDNA was synthesised from 100 ng of the *P. ovis *aRNA samples using Superscript II (Invitrogen, UK) and oligo(dT) primers (Sigma-Aldrich, UK) according to manufacturer's instructions. qPCR was performed in quadruplicate on cDNA samples using an ABI Prism 7000 real-time thermal cycler (Applied Biosystems, UK) and the primer sets as defined above. Standard curves were constructed from serial 10-fold dilutions (10^8^-10^2 ^copies per μl) of previously constructed and linearised plasmids containing the relevant *P. ovis *gene (Pso o 1, Pso o 2 or beta-actin) and amplified in parallel with each series of samples allowing the automatic generation of standard curves using the Applied Biosystems 7000 System SDS software. Correlation co-efficients of the standard curves were between 0.97-0.99 and PCR efficiencies calculated from the slopes were ≥90%. The number of copies per microlitre of cDNA was calculated and the results normalised to that of *P. ovis *beta-actin.

## Results

### Microarray construction and gene products represented on the microarray

The most abundant phylum associated with the top BLAST hits for the ESTs used in the construction of the array was the arthropoda, with the most common representative species being the deer tick, *Ixodes scapularis *(172 ESTs or 18% of the total with significant BLAST hits). This probably reflects the observation that *I. scapularis *represents the most closely related species to *P. ovis *with an annotated genome. In addition, a number of ESTs shared homology with gene products from the closely related HDMs *D. pteronyssinus *(10 ESTs) and *D. farinae *(8 ESTs), including homologues of mite allergens, i.e. Der p 1, Der p 2, Der p 3 and Der p 21. Annotation of the *P. ovis *cDNA microarray can be seen in Additional File 1.

### Gene ontology (GO) annotation of the microarray

The sequences of the probes on the array cover a total of 3,509 individual GO terms and GO term annotation showed that 644 of the 981 microarray probes (67%) were associated with at least 1 GO term, with 89 probes being associated with > 10 GO terms. These GO associations can be broken down into the major categories of biological process (43%), molecular function (31%) and cellular component (26%). All EST sequences represented on the array have been submitted to GenBank and the relevant accession numbers associated with each probe are denoted in Additional File 1. In addition the full GO annotation of the *P. ovis *ESTs used for the construction of the cDNA microarray has been published previously [17].

### Microarray performance

**Figure 1:**
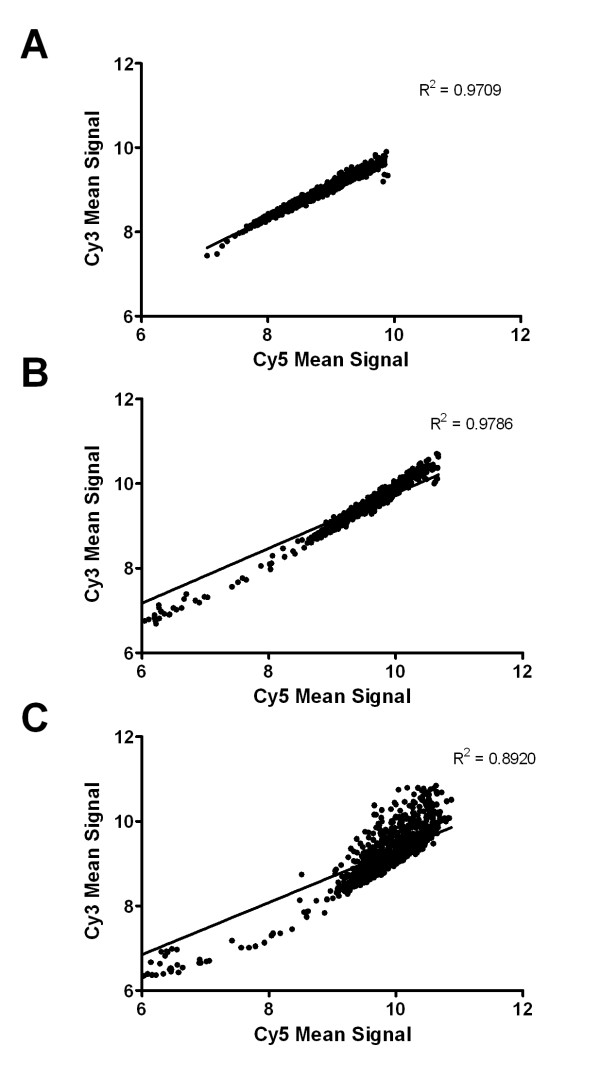
**Scatter plots demonstrating the degree of variance between different sample hybridisations**. Scatter plots were constructed from the raw non-normalised, transformed (natural log) array data, averaged for each comparison from the four hybridisations (2 dye-swapped (Cy3/Cy5) replicates each). **A**. Self *vs *self hybridisation (Fed *P. ovis *control *vs *fed *P. ovis *control). **B**. Biological replicates (replicated analysis of fed *P. ovis *control *vs *starved *P. ovis*, compared using two separate dye swapped arrays). **C**. Activated sample *vs *control sample (starved *P. ovis vs *fed *P. ovis *control). Scatter plots, linear regression analysis and corresponding R^2 ^values were generated in GraphPad Prism (GraphPad Software Inc, Version 5.04).

To assess the performance of the array the following dye-swapped hybridisation comparisons were set up: **A**. Self (fed *P. ovis*) *vs *self (fed *P. ovis*), **B**. Biological replicates (replicated analysis of fed *P. ovis *control *vs *starved *P. ovis*, compared using two separate dye swapped arrays), **C**. Activated biological sample (starved *P. ovis*) vs control sample (fed *P. ovis*). Scatter plots demonstrating the degree of variance between each of these comparisons can be seen in Figure 1. These demonstrate the high degree of correlation between the self *vs *self hybridisations (R^2 ^= 0.9709) and between replicate samples (R^2 ^= 0.9786), whilst also showing the increased level of variation within the biological comparison, i.e. fed *P. ovis vs *starved *P. ovis *mites (R^2 ^= 0.8920).

### Experimental Design

Array performance on biological samples was assessed through the comparison of RNA from "fed" *P. ovis *mites compared with RNA from *P. ovis *mites, which had been "starved" for 4 days. Samples were prepared as described above and analysed in duplicate with dye swapped (Cy3/Cy5) samples included, i.e. four hybridisations each.

### Fed *vs *starved *P. ovis *comparison

Based on the parameters described above (FDR ≤0.05 and FC ≥1.5) a total of 684 probes represented transcripts which were differentially expressed between the fed and starved *P. ovis *mite samples. Nine of these probes represented transcripts which were present at higher levels in the fed mites (FC range from 1.51 - 2.47) whilst the remaining 675 probes represented transcripts which were present at lower levels in fed mites (i.e. higher levels in starved mites) FC range 1.5 - 2.68.

### Fed *P. ovis - *enriched transcripts

Transcripts present at higher levels in the fed mites included well-characterised mite allergens; the cysteine protease Pso o 1 (FC = 2.17), the gut associated factor Pso o 2 (FC = 2.15) and a *P. ovis *homologue of the HDM group 21 allergen (Der p 21), termed here Pso o 21 (FC = 1.83). The full list of transcripts found to be up-regulated in the fed *P. ovis *mite sample can be seen in Table 1.

**Table 1:** Transcripts significantly up-regulated in the fed *P.ovis *mite sample

GenBank accession number	Probe ID	Sequence description	Homologous species	Minimum E-value	Fold change (fed *P. ovis vs *starved *P. ovis*)	FDR corrected p-value
FR748349	Bu_002_g12	-	-	-	1.55	0.0017
FR748466	Bu_004_b02	Beta-1,3-D-glucanase	*Cryptopygus antarcticus*	8.82E-22	2.32	0.00002
FR748550	Bu_005_a09	-	-	-	2.47	0.002
FR748633	Bu_005_h12	Pso o 1/Der p 1 allergen	*P. ovis/D. pteronyssinus*	2.36E-46	2.17	0.00001
FR748686	Bu_006_e09	Macrolide glycosyltransferase	*Streptosporangium roseum*	1.49E-44	1.95	0.00002
FR748740	Bu_007_b11	Der p 21 allergen	*D. farinae*	2.13E-36	1.82	0.0002
-	Bu_007_e02	-	-	-	1.51	0.0002
FR748952	Bu_010_f10	Myosin light chain	*Haemaphysalis qinghaiensis*	3.26E-57	1.53	0.0029
FR749449	Bu_017_f04	Pso o 2 group 2 allergen	*P. ovis*	1.49E-53	2.15	0.0025

Probe ID represents the unique identifier for the microarray probe. FDR corrected p-value shows the Benjamini & Hochberg False Discovery Rate (FDR) corrected p-value for each transcript [23]. E-value is the Expect value which describes the number of BLAST hits one can expect to see by chance for a given sequence, the lower the E-value the more significant the match.

### Starved *P. ovis *- enriched transcripts

Among transcripts present at higher levels in the starved mites were homologues of those encoding five heat shock proteins, three of which were homologues of putative proteins from the deer tick, *I. scapularis*. We also observed the 2-fold up-regulation of a homologue of the receptor (chemosensory) transporting protein from *I. scapularis *and receptor accessory protein 4 from *Caenorhabditis briggsae *in starved *P. ovis *mites. Homologues of two genes with roles in autophagy [autophagy related homologue 3 (ATG3) and DNA-damage regulated autophagy modulator 1 (DRAM1)] also showed 2-fold up-regulation in the starved mites. The top 20 transcripts (highest fold change) up-regulated in the starved *P. ovis *mite sample can be seen in Table 2 and the full list of 675 probes is available in Additional File 2.

**Table 2:** Top 20 transcripts significantly up-regulated in the starved *P.ovis *mite sample.

GenBank accession number	Probe ID	Sequence description	Homologous species	Minimum E-value	Fold change (starved *P. ovis vs *fed *P. ovis*)	FDR corrected p-value
FR748352	Bu_002_h03	Calcium-independent phospholipase A2-gamma	*Tribolium castaneum*	1.6E-49	2.68	1.45E-05
FR749255	Bu_015_c09	Heat shock protein HSP90-alpha	*Ixodes scapularis*	5.67E-77	2.66	0.015
**-**	Bu_016_f12	-	-	-	2.61	1.93E-05
FR749314	Bu_016_a06	Lipase, family member K/J	*Pan troglodytes*	2.93E-37	2.56	2.62E-07
FR749136	Bu_013_g02	Ras-related GTP binding A	*I. scapularis*	6.02E-46	2.5	2.79E-05
FR748742	Bu_007_c01	ABC transporter	*I. scapularis*	6.48E-62	2.47	1.54E-05
FR748556	Bu_005_b04	-	-	-	2.46	1.49E-05
**-**	Bu_014_d11	-	-	-	2.44	0.002635
FR749256	Bu_015_c10	Solute carrier family 27 (fatty acid transporter)	*Nasonia vitripennis*	4.01E-40	2.43	0.00018
FR749382	Bu_016_h02	RNA-binding protein MEX3C-like	*Drosophila virilis*	0.00016	2.42	2.59E-08
FR749562	Bu_011_a04	Tubulin beta-4 chain-like	*Caernorhabditis briggsae*	1.46E-24	2.41	8.22E-05
FR748807	Bu_009_a04	Polo-like kinase 3	*Nematostella vectensis*	5.49E-16	2.39	0.005
FR749241	Bu_015_b06	MAD2 mitotic arrest deficient-like 1	*Branchiostoma floridae*	4.09E-50	2.39	8.73E-05
FR749251	Bu_015_c05	Replication factor C subunit 5	*Acyrthosiphon pisum*	1.49E-89	2.39	0.0001
FR749291	Bu_015_f12	Sporozoite asparagine-rich protein	*Plasmodium falciparum*	4E-04	2.36	0.0016
FR748903	Bu_010_b08	Exosome component 7	*I. scapularis*	6.59E-41	2.33	0.001273
FR748544	Bu_005_a03	-	-	-	2.32	3.73E-08
FR749434	Bu_017_d11	Hydroxysteroid (17-beta) dehydrogenase	*I. scapularis*	1.77E-13	2.3	0.000138
FR749176	Bu_014_c08	-	-	-	2.29	0.000162
FR749632	Bu_011_g07	Ankyrin repeat domain-containing protein 49	*Saccoglossus kowalevskii*	2.52E-32	2.29	0.004882

Probe ID represents the unique identifier for the microarray probe. FDR corrected p-value shows the Benjamini & Hochberg False Discovery Rate (FDR) corrected p-value for each transcript [23]. E-value is the Expect value which describes the number of BLAST hits one can expect to see by chance for a given sequence, the lower the E-value the more significant the match.

### qPCR analysis of selected microarray probes

The differential expression of two selected genes (Pso o 1 and Pso o 2) between fed and starved *P. ovis *mites was verified using qPCR, which confirmed the up-regulation of both genes in fed *P. ovis *mites. The microarray data highlighted an approximate 2-fold up-regulation of both Pso o 1 and Pso o 2 in fed *P. ovis *mites and the qPCR results for these genes further supported these findings with the up-regulation of both genes being confirmed. The magnitude of change in the level of Pso o 2 was very similar between the two techniques, with a 3.1-fold change seen in the qPCR (compared to 2.15-fold with the microarray), whilst the magnitude of the change in Pso o 1 was higher with the qPCR at 9.6-fold (compared to 2.17-fold with the microarray). Therefore, this analysis demonstrated a good degree of agreement between the qPCR and mite cDNA array data between fed *vs *starved *P. ovis *mites.

## Discussion

Here we have described the development, construction and utilisation of a cDNA microarray based on the currently available ESTs for the economically important ectoparasite, *P. ovis*. This currently represents the most extensive transcriptomic resource for the analysis of this parasite. The *P. ovis *probes on the microarray were derived from a mixed population of mites including larvae, nymphs, and male and ovigerous female adults. As such these are likely to represent a broad range of expressed genes across all *P. ovis *life cycle stages, producing an array that is applicable to a range of different analyses. Sufficient controls were included in the array design to ensure effective normalisation and all of the EST probes were printed in triplicate enabling an accurate estimation of gene expression. The content of the array, although limited to the currently available *P. ovis *ESTs, includes homologues of HDM allergens and probes for a number of other important mite factors, i.e. proteolytic enzymes, homologues of tick salivary factors, heat shock proteins and factors involved in drug and xenobiotic metabolism [17]. In addition, the array contains factors with no significant homology from BLAST searching which may represent novel *P. ovis *transcripts and thus provides the opportunity for novel discoveries into the underlying biology of the mite.

By using this microarray to analyse the expression of suites of genes relating to a fundamental feature of host:parasite interaction and mite biology (i.e. feeding), we are moving towards the rational selection of molecular targets for parasite intervention - the identification of vaccine candidates by a "rational" approach. This approach, based on an understanding of: i) which molecules are essential to survival, and ii) the accessibility of these molecules to the host immune system, has been advocated in the field of ectoparasite vaccine development for several years [25]. Thus far, for *P. ovis*, candidate antigens have been identified through either a "pragmatic" approach of fractionating native protein extracts of the mites and using these as vaccines [15,16], or by immunoscreening cDNA libraries [18]. The use of a *P. ovis *microarray represents an alternative approach, by targeting those molecules which are differentially regulated during, for example, feeding we can start to address the first of the criteria for rational selection.

The genes up-regulated in the fed mites included homologues of the well characterised HDM allergens Der p 1 and Der p 2, namely Pso o 1 and Pso o 2. Both factors are known to be expressed in the mite gut and are hypothesised to play key roles in mite digestive processes [26,27]. In further support of these findings a previous study using the method of suppressive subtractive hybridisation demonstrated the up-regulation of Pso o 1 in fed *vs *starved *P. ovis *mites [28]. Pso o 2 is a homologue of the HDM allergen Der p 2 which has been shown to act as a functional mimic of the toll-like receptor 4 (TLR4) accessory protein; MD-2 [29]. Der p 2 has also been localised to mite gut and faecal pellets and may also be involved in the triggering of the cutaneous inflammatory response upon which the mites feed [8,9]. The allergen Der p 21 (Pso o 21 in *P. ovis*) has also been localised to HDM gut and faecal pellets and may have a role in mite feeding [30] and up-regulation of this transcript in fed *P. ovis *mites may be indicative of active digestion. These allergens have previously been localised to HDM faecal pellets and their homologues in *P. ovis *are therefore likely to come into contact with host skin [26,27,30]. During infestation, *P. ovis *does not penetrate beyond the stratum corneum and the host cutaneous inflammatory response appears to be triggered by the presence of proteolytic enzymes, such as Pso o 1. The observation that these allergens are recognised by the host immune response following infestation suggests they may be valid targets for immunisation [26,30].

Amongst the transcripts up-regulated in starved mites were a number representing genes involved in the stress response, including those implicated in the cellular response to heat shock. Heat shock proteins are molecular chaperones with key roles in signal transduction, protein folding and degradation and can also be up-regulated as part of the stress response to starvation [31,32]. Homologues of two tick genes involved in autophagy were also up-regulated in starved *P. ovis *mites, namely ATG3 and DRAM1. The process of autophagy is a critical proteolysis system that is induced during starvation [33]. Although the *P. ovis *life cycle takes place in its entirety on the host, the mites are able to survive off-host for up 15-16 days and they, like other arthropods, must therefore possess a degree of tolerance to starvation [4,10]. In ticks the process of autophagy helps to compensate for the lack of host-derived nutrients during periods of time spent off-host [34,35]. The identification of genes potentially involved in autophagy indicates that a similar process may also exist in *P. ovis*, thus protecting mites during periods spent off-host and allowing mites to survive during transfer from one host to the next. Also of note was the 2-fold up-regulation of a homologue of the receptor (chemosensory) transporting protein from *I. scapularis *and receptor accessory protein 4 from *Caenorhabditis briggsae *in starved *P. ovis *mites. These factors have been implicated in the promotion of the functional cell surface expression of odorant receptors [36] which play key roles in the detection of odour and in the location of the host or a preferred food source [37]. The up-regulation of these factors in starved mites may be indicative of the mite response to a lack of nutrients by increasing efforts to locate a food source. This is further supported by the previous identification of an olfactory receptor up-regulated in starved *P. ovis *mites [28]. Also up-regulated in starved mites were two probes representing homologues of *I. scapularis *secreted salivary factors (a secreted salivary gland peptide (2-fold) and a secreted salivary protein (1.6-fold)). Although salivary gland structures have not been conclusively identified in *P. ovis *they are present in other Astigmatid mites e.g. *Acarus siro *[38]. In addition four homologues of potential salivary/oesophageal gland factors have been identified in *P. ovis*, although in this study these factors were up-regulated in fed mites [28]. The up-regulation of salivary factors in starved mites as described here may be indicative of increased preparation to feed and the observed contradiction in regulation between this and the previous study [28], is likely to be due to the identification of different factors in each study.

## Conclusions

Through the analysis described in this study, the *P. ovis *array has been shown to be robust and more importantly to provide highly reproducible results. Although the emerging technologies of next generation sequencing and digital transcriptomic analysis are set to transform this type of analysis in the future, they currently require the presence of a genome or transcriptome upon which to map digital sequence reads. In addition, each separate comparison requires that an entirely new analysis be performed, meaning that the cost is replicated on each occasion. In contrast, the array described here enables multiple comparisons to be made for a relatively low cost, i.e. the nominal cost of a printed slide and reagents. The *P. ovis *array provides an excellent resource for the transcriptomic analysis of *P. ovis *and related mites and opens up the possibility of future studies to dissect as yet unexplored aspects of mite virulence [39], the increasing problem of drug resistance [40,41] and the transcriptional analysis of different life cycles stages, i.e. egg, larva, nymphal stages and adults. The *P. ovis *cDNA array is available from the authors upon request.

## Competing interests

The authors declare that they have no competing interests.

## Authors' contributions

STGB designed the study, performed and processed mite samples and RNA extractions, participated in the design and annotation of the microarray, analysed the microarray and qPCR data and wrote the manuscript. AD participated in the study design, constructed the *P. ovis *microarray, processed the samples and microarray hybridisations and analysed the microarray data. CAW participated in the microarray design, annotation and EBI submission of sequence and microarray data and helped to prepare the manuscript. EJM performed and analysed the qPCR comparisons. AJN participated in the study design and data analysis and helped to prepare the manuscript. FK provided access to *P. ovis *expressed sequence tags and assisted with data analysis and manuscript preparation. CM assisted with data analysis and manuscript preparation and supplied sequence data and constructs for Pso o 1 and Pso o 2. JFH conceived and designed the study and helped to prepare the manuscript. All authors have read and approved the manuscript.

## Supplementary Material

Additional file 1 ***P. ovis *cDNA microarray annotation data**. This file contains the annotation of the probes used in the construction of the *P. ovis *cDNA microarray, which includes Probe ID, GenBank accession ID and sequence description from BLAST analysis. Click here for file

Additional file 2 **Microarray transcripts up-regulated in starved *P. ovis *mites**. This file contains the full list of 675 probes found to be significantly up-regulated in starved *P. ovis *mites and contains the Probe ID, GenBank accession ID, sequence description, minimum E-value, fold change and FDR corrected p-value for each probe. Click here for file
